# Community-level women’s education and undernutrition among Indian adolescents: A multilevel analysis of a national survey

**DOI:** 10.1371/journal.pone.0251427

**Published:** 2021-05-20

**Authors:** Ankita R. Shah, Malavika A. Subramanyam

**Affiliations:** Social Epidemiology, IIT Gandhinagar, Gandhinagar, India; University of Mississippi Medical Center, UNITED STATES

## Abstract

**Background:**

Little research has explored the influence of social context on health of Indian adolescents. We conceptualized community-level women’s education (proxy for value placed on women’s wellbeing) as exerting contextual influence on adolescent hemoglobin level and body mass index (BMI).

**Methods:**

We derived our sample of more than 62,000 adolescent aged 15 through 17 years from the Indian National Family Health Survey 2015–16. The sample consisted of a total of 62648 adolescents (54232 girls and 8416 boys) for the hemoglobin, and 62846 adolescents (54383 girls and 8463 boys) for the BMI analysis. We fitted multilevel random intercepts linear regression models to test the association of village- and urban-ward-level-women’s education with hemoglobin level and BMI of adolescents, accounting for their own and their mother’s education; as well as relevant covariates.

**Findings:**

Our fully adjusted model estimated that if the 52% of communities with less than 20 percent of women having a tenth-grade education in our sample were to achieve 100 percent tenth-grade completion in women, hemoglobin would be 0·2 g/dl higher (p<0·001) and BMI would be 0·62 kg/m^2^ higher on average among *all adolescents* in such communities. Unexplained variance estimates at the contextual level remained statistically significant, indicating the importance of context on adolescent undernutrition.

**Interpretations:**

Adolescents are deeply embedded in their context, influenced by contextual factors affecting health. Promoting adolescent health therefore implies altering social norms related to adolescent health and health behaviors; along with structural changes creating a health-promoting environment. Integrating our empirical findings with theoretically plausible pathways connecting community-level women’s education with adolescent undernutrition, we suggest that enhancing community-level women’s education beyond high school is necessary to facilitate these processes.

**Implications:**

Addressing contextual determinants of adolescent undernutrition might be the missing link in India’s adolescent anemia and undernutrition prevention efforts, which are currently focused heavily on individual-level biomedical determinants of the problem.

## Introduction

Adolescence (10 to 19 years) is a period of rapid physical growth in human development, substantially increasing nutritional demand [1]. This period is crucial as it offers a second chance to correct the nutritional deficiencies that might have occurred earlier; as well as to sustain the gains in nutrition achieved during childhood [2]. Unfortunately, different forms of undernutrition are highly prevalent among adolescents across low- and lower-middle-income countries, including India [3]. Low levels of hemoglobin (or anemia) and underweight are among the pressing health risks in Indian adolescents and demand urgent action [4].

With 253 million adolescents, roughly a quarter of its population, India is home to the world’s largest number of adolescents [5]. Anemia among Indian adolescents affects 40% of girls and 18% of boys [5]. Thinness which indicates short-term undernutrition affects 18% of adolescent girls and 29% of adolescent boys in India [5]. Anemia and other forms of undernutrition severely curb growth and cognitive development, adversely affect productivity, and increase the risk of poor pregnancy outcomes among adolescent mothers, thus perpetuating the intergenerational cycle of undernutrition [5]. Despite such severe impacts, there is a paucity of research on undernutrition, including low hemoglobin levels and underweight, in Indian adolescents, that focuses on the influence of determinants beyond the individual level [5]. We argue that this is suggestive of a lack of imagination in the theoretical lens employed.

While a range of individual-level social, behavioral, biological and physiological risk factors associated with anemia [6, 7] and underweight [8] in adolescent girls are reported, there is scant research examining the potential impact of context on undernutrition among adolescents. We conceptualize that living in neighborhoods that support the sociocultural norms valuing women’s well-being would promote adolescent health. We investigate this by quantifying the association of community-level women’s education with adolescent hemoglobin level, and body mass index (BMI), above and beyond the role of their mother’s education.

### Theoretical premise

The theoretical foundation of conceptualizing the impact of community-level women’s education on adolescent health is rooted in the ecosocial theory [9]. The theory cautions against a simplistic understanding of determinants of disease (and health) at the individual level. It views individuals as non-separable from society because social and biological determinants are intertwined at every level: Individual, family, community or neighborhoods, regional, national and global [9]. It, therefore, encourages epidemiologists to inquire into the social context of health using a multilevel perspective [9]. Drawing from this theory, we conceptualized adolescents as deeply embedded in their context and, by extension, the unequal distribution of low hemoglobin levels and undernutrition among them as an embodiment of their lived social environment. Extant literature has pointed out that socio-economic and political context, including cultural and societal values, social stratification based on gender, caste, class, and religion, are important social determinants of nutrition in adolescents [10]. These social determinants operate at global, regional, and local levels through complex intersecting pathways, determining the access to, and availability of, food, health, education, and security to adolescents [10]. An individual-focused perspective fails to take into account the causal influence of the context on adolescents’ undernutrition [9]. Exploring and addressing the contextual determinants of adolescent health is thus essential in addressing this stubborn problem. We argue that the value assigned to women in society is an integral aspect of the social context within which adolescents live and grow, acquire skills and education, and often work. We posit that this has relevance to adolescents’ health and nutritional status.

Patriarchal norms are deeply pervasive in Indian society with its manifestations and effects varying depending on socio-economic and regional contexts [11–13]. The lower value assigned to women as compared to men in Indian society is evident in a range of socio-economic and developmental indicators. For instance, India was ranked 112^th^ among 153 countries in the World Economic Forum’s Global Gender Gap Index, 2019–2020 [14]. Discrimination against women begins even before birth in India as evidenced by an inverse sex ratio—according to the latest estimates, 899 girls are born for every 1000 boys born [15]. This inverse sex ratio at birth has been argued to primarily result from gender-biased sex-selection practices rooted in son-preference [16, 17]. Evidence suggests that gender discrimination continues after birth with the neglect of the girl child—11% more girls than boys die under the age of five [18]. This is in stark contrast to the world average, where 7% more boys, than girls, dies under the age of five [18]. According to UNICEF, India is the only large country in the world that has an inverse sex ratio at birth accompanied by postnatal sex selection [18]. With 460 thousand girls missing every year between 2013 to 2017, India accounted for nearly one third of the world’s missing women as of 2020 [17]. India is also home to the world’s highest number of child brides [18]. Although child marriage in India is showing a declining trend, the rate remains high at 27% [19]. Further, nearly 27 percent married adolescents reported to have given birth to one child [20]. This has huge health and economic consequences on the adolescent herself, her off-spring, and the family [21]. Notably, female labor force participation in India is among the lowest in the world: 2020 data show that four out of five women were not working for wages [22]. This is lower than the average values for lower-middle income countries and the South Asian region. Indian women are also disadvantaged in ownership of assets and access to healthcare [19]. Moreover, women are commonly exposed to gender-based violence in India, with the latest nationally representative data suggesting that 33% of ever-married women between 15 to 49 years were exposed to at least one incidence of physical, sexual or emotional spousal violence [19].

While historically poor investment in girls’ education has resulted in an overall gender gap in literacy, India is rapidly making progress on closing the gender gap in literacy among children [23], and secondary school enrolment rates [24]. However, the gender gap in education among the socio-economically marginalized groups is still a problem [25, 26]. The gender gap in completion of secondary schooling (tenth-grade) also persists, likely because of the gender bias favoring boys regarding an investment in education [27] and the provision of free education only until the age of fourteen under the Right of Children to Free and Compulsory Education act, 2009, popularly known as Right to Education (RTE) Act, 2009 [28]. According to the Indian National Sample Survey of 2018, the literacy rate (percentage among persons of age 7 years and above) in men is 81.5% versus 65% in women [29]. The same source indicated that the percentage of persons not literate in those aged 15 years and above was 22.2% among men and 41.2% among women; while the proportion who had completed some primary schooling (grades 1 to 4 was 21.2% among men and 20.4% among women; -middle school (grades 5 to 7) was 19.8% among men and 14.4% among women;—secondary school (grade 8 to 10) was 17.3% among men and 12.5% among women;—higher secondary (grades 11 to 12) was 12% among men and 7.6% among women; and -graduate or above was 7.4% among men and 3.9% among women [29].

In this backdrop, we conceptualized community-level women’s education as a proxy for the value that a community places on women’s well-being. This is based on the assumption that societies where women are valued, ‘as women’, tend to have a greater access to education for women [30]. We recognize that women’s education is a result of the amalgamation of a range of structural, socio-cultural, and household-level factors [31]. We posit that societies that value their women, see the value in educating their women, and in increasing the social desirability and social acceptability of women getting an education. We also surmise that these societies tend to invest in, and generate resources to facilitate, women’s access to education. Thus, we argue, one feature of societies that value their women could be a greater proportion of women with higher levels of education.

We have not empirically examined the potential pathways through which the contextual effect of the value assigned to women’s wellbeing in a community manifest. However, contextual effects and diffusion of behaviors, knowledge and values have been conceptualized in several disciplines, for instance in political science [32], in demography and fertility transition [33] as well as in public health such as neighborhoods and health [34] and health behavior change [35]. Drawing from these scholars as well as the ecosocial theory [9], we conceptualized the pathways linking the contextual influences of the value assigned to women’s wellbeing in a community on adolescent nutrition.

Communities where women are valued are likely to assign a higher value to women’s knowledge, skills, and abilities [30, 36]. Women in these communities might have a higher self-worth and confidence (intangible resources) to assert their preferences and be able to better leverage or even generate material resources to enact their choices [30]. These changes could alter the social norms in a community and influence the decision-making of adolescents in those communities. Women in such communities might have a greater say in decision making within the household as well as in the public sphere, such that they are able to negotiate the use of resources and the benefits accruing from their use, to achieve the goals that they value, which likely includes the betterment of the nutritional status of adolescents in their communities [30, 37]. Women in influential positions in communities have the potential to transform social institutions and promote the growth of public services, including health and nutrition services in a community [37]. All of these have the potential to impact adolescent nutrition. Extant literature has suggested that often women are endowed with primary care-giving responsibility within the household [38]. Women also have a central role in maintaining household food security and household health environment [38]. In such a context, the influence of women’s empowerment and/or education on child health has suggested that women who have access to resources and a greater say in decision-making tend to prioritize pro-child resource allocation such as towards food, health care, and education [37–39]. Extending this to adolescent children, *we argue that women’s health-conducive and -progressive behaviors related to adolescent health*, *nutrition*, *education*, *sanitation and hygiene*, *diffuse within the community*, *through social interactions*. This potentially alters the normative expectations within the community and reinforces health-conducive norms and practices among family members of the adolescents, regardless of their own individual or family characteristics. This could be illustrated by a thought exercise where we compare two families with similar socio-economic backgrounds, having two adolescent children (a boy and a girl). Say family A lives in an area where generally women are valued as opposed to a family B living in a community where women are less valued. Family A would have a higher chance of exposure to health-enhancing choices made by a larger proportion of empowered women in the neighborhood. Social interaction among them likely provides an opportunity for social learning and social influence, potentially reinforcing health-promoting practices and norms. This might alter family-A’s beliefs and practices related to adolescent care, benefitting adolescents in family-A, irrespective of the status of women in family-A. Such an effect might operate through women, men and/or adolescents themselves within family-A. Adolescents in family-A might also benefit from being less exposed to gender-unequal social norms in their social environment, which might promote their self-worth and confidence that may subsequently help them negotiate gender-equal situations within a familial setting. On the other hand, family-B has a smaller chance of social interaction with women who make health-promoting choices, as the proportion of such women in their community is lower. Therefore, the social pressure to reinforce health-promoting behaviors is likely much lower. Adolescents in family-B therefore might not benefit from a health-promoting contextual effect of living in a community that values women unlike adolescents in family-A. Furthermore, adolescents growing up in a context that is more gender unequal, will more likely be imbued with those values, resulting in their own lower self-worth and lower confidence to assert their choices in a familial setting, a potentially more direct impact for adolescent girls. This might manifest in their lower ability to bargain for nutrition- or health-related resources and social support, for instance: consuming nutritionally-dense food, getting access to health-care or access to hygienic environments, products and practices especially related to menstruation.

To summarize, despite being theorized, our extensive literature search could not locate any theory-driven research that empirically demonstrated the influence of context on the hemoglobin level, anemia, nutritional status, or health of adolescents, in low- or lower-middle-income countries, including India. To our knowledge, this is the first study that investigates the relationship of community-level women’s education with adolescents’ nutritional status. Notably, our interest in community-level women’s education is not limited to its immediate exposure. Rather, we conceptualize it as capturing the strength of the socio-cultural norms in the community that value women’s well-being. Thus, our inquiry has relevance not only for Indian adolescents, but might have implications for the millions of adolescents growing up in societies that are embedded in patriarchy.

## Methods

### Dataset and sample characteristics

This analysis is based on publicly available, de-identified data from the fourth round of the National Family Health Survey (NFHS-4), India, undertaken in 2015–16 [40]. It was a nationally representative survey yielding estimates at the district and state levels [19]. The sampling design treated villages (occasionally village-clusters) and census-enumeration wards (in urban areas) as the Primary Sampling Units (PSUs) [19].

In the present study we have operationalized the PSU as the “community”. The sample households in the clusters (PSUs) are a mix of different caste-groups residing in the same neighborhood in both urban as well as rural areas. However, the proportion of households of different caste groups across clusters might vary depending on the caste composition of the cluster. Our operationalization of the community therefore captures geographic proximity of the households. The sample population might be conceptualized as living in the same neighborhoods. Physical proximity of residences and shared interests related to availability of public infrastructure typically facilitate social interaction among the residents. In the rural areas, the villages or cluster of villages are frequently the part of the same local government (Gram Panchayat). Social interactions might be further strengthened by the involvement of women in local self-help groups (women’s microfinance groups) or children sharing the same pre-school (Anganwadi). Similar opportunities for greater social interaction and the development of a sense of community are observed even in urban areas. While the CEBs are spaces with relatively higher population density, they often have shared sources of water, shared public toilets and other facilities such as Anganwadi centers. They might also share geographic access to markets, infrastructure, and services. All this might promote social interaction among the local residents. Thus, PSUs might not only serve as a delimited geographical and/or administrative region. It is likely viewed by the residents as a social space with a sense of belonging in the form of ‘our village’ or ‘our place’ or ‘our neighbors.’ However, other factors influencing social interaction such as caste and religious dynamics do not get adequately captured in this operationalization of the community, which remains a limitation, even though a sample from a PSU includes households having different caste and religious characteristics. Conceptually, neighborhoods have emerged as potentially relevant in public health as they capture the social and physical aspects of contexts that have theoretically plausible causal association with health [34]. Scholars have previously operationalized PSUs as communities while estimating the impact of social characteristics of neighborhoods on health outcomes in the Indian context [39]. A total of 601,509 households were included in NFHS-4, which were selected by employing a two-stage stratified random sampling design [19], (for additional details on sampling design refer [19]) from 640 districts of all the 36 states/union territories (UT) in India. Hemoglobin was measured in a total of 117,711 girls and 17,912 boys aged 15 through 19 years. We restricted our analysis to unmarried adolescents between 15 through 17 years of age, as information on parents’ education was available only for children below 18 years of age [19]. We linked the hemoglobin levels of 75,696 adolescents to their mother’s education. Information on father’s education was collected only if the father permanently lived in the same household as that of the adolescents [19] (information not available for instance if father had migrated for work). Therefore, we excluded 11,106 adolescents who were missing the information on their father’s education. After excluding observations that were missing data (including the response “don’t know”) on any of the other covariates, our analytical sample size was 62,648 adolescents for the hemoglobin analysis. Following a similar process, of 18,984 boys and 1,25,333 girls aged 15 through 19 whose body mass index (BMI) was measured, we linked the BMI of 75944 adolescents to their mother’s education. After deleting observations with missing data on covariates we obtained an analytical sample of 62,846 adolescents aged 15 through 17 years for the BMI analysis.

### Measures

The outcome variables were blood hemoglobin level in adolescents measured in grams per deciliter (g/dl), and BMI measured as weight in kilograms divided by the square of height in meters (kg/m^2^) on continuous ratio scales, excluding biologically implausible values [41, 42]. Hemoglobin estimation was conducted on-site with a battery-operated portable HemoCue Hb 201+ analyser [19]. Hemoglobin levels were adjusted for smoking and altitude in areas that were above 1,000 meters [19]. Weight and height were measured on-site using a Seca 874 digital scale and a Seca 213 stadiometer respectively [19].

Our main predictor was the education of women at the community-level, represented as the proportion of all women in NFHS-4 above 19 years of age in a PSU who had completed formal education at least till the tenth-grade. The education data of a total of 880,431 women across 28524 PSUs were used to compute this variable. NFHS-4 collected information about the education level of each family member of the household above the age of four years. We therefore had information about women’s education for 1,041,971 women in total. After removing those aged below 19 years, we had information from a total of 880,850 women above 19 years of age. Further removing those who were missing data on education yielded data on the education of women above 19 years of age from a total of 880, 431 women. This information was utilized to compute the community-level women’s education variable.

Our selection of a tenth-grade education for operationalizing community-level women’s education is based on the idea that the completion of a tenth-grade education among women involves valuing women’s education beyond the elementary level (Grades 1 to 8), even when it is not compulsory and free for the children under the RTE Act, 2009 [28]. Further, historically, completion of the tenth grade is an important milestone in the Indian education system [26]. One of the reasons for this is that it involves clearing an examination that is conducted by either a central or a state secondary school examination board [26]. This examination requires students to appear for the exam at a place that is centrally decided, often different from their own school, sometimes in a different village or town than their own. In this standard examination, a student’s academic performance is assessed by external evaluators. There is no such requirement for any lower grades. Furthermore, after the introduction of the Right to Education Act 2009, a no-detention policy until grade 8 was implemented in India [28]. In many schools, this likely resulted, unintentionally, in an improper assessment of children in elementary school [43]. This is described as one of the reasons why children who were poorly assessed in the elementary grades tended to lose interest in studies when they performed poorly at higher grades [44]. Despite the several critiques of the board examination [45], we believe that for the above-mentioned reasons, this examination might serve as a tool to assess with greater integrity the academic potential of a student. This implies that we expect a student who has passed the tenth-grade to manifest a greater level of the desirable impacts of schooling such as critical thinking and a progressive attitude towards change in social norms than students who have basic numeracy and literacy skills. Further, the tenth-grade examination plays a decisive role in making further education opportunities available to the child in the Indian education system [26]. Passing a tenth-grade exam carries a value in the labor market as well, opening up several job opportunities for the candidate (for instance in one of the greatly sought-after employment sectors, the government [46]). In India, secondary schooling is reported to be giving an 11.4% rate of return to education for every additional year of schooling at the secondary schooling level, as compared to the same for primary and middle school at 5.5% and 6.2% respectively [47]. On the other hand, the rate of return to education for every additional year of schooling at higher-secondary and graduate levels are 12.2% and 15.9% respectively [47]. Thus, the rate of return to schooling is reported to be much higher for completion of secondary schooling in comparison to primary and middle school. Additionally, one of the major points at which substantial dropout from school is observed in India is between grades 9 and 11 [48]. Considering these points, we believe that for the purpose of the present study, the tenth-grade serves as an appropriate cut-off to measure the importance given to women’s education and, by extension, we argue, the importance given to women themselves, in a community.

We used the following variables as covariates in our analysis. We operationalized the age of adolescents (in years), education of adolescents, adolescent’s mother, and adolescent’s father (all three as years of formal schooling completed), as well as household size (as the number of family members) as continuous variables. We operationalized gender (girls or boys, reference = boys), religion (Hindu, Muslim, Christian, and others; reference = Hindu), social group (Scheduled Tribe-ST, Scheduled Caste-SC, other backward classes- OBC, and general; reference = SC), household wealth (as quintiles of the distribution of the population on a household asset score included in the original dataset; the five quintiles were poorest, poorer, middle, richer or richest; reference = poorest), place of residence (urban or rural; reference = urban), and sub-national regions (groups of states in the North, East, West, Central, South and Northeast; reference = North) as categorical variables. Social groups denoted as SC, ST or OBC are historically socially and economically marginalized caste groups in India as recognized by the Government of India. These labels are employed by the Government of India in official documents and widely used in daily life in the Indian context. The wealth quintile data that we have used were provided in the original dataset [19, 40] (for further details on how the wealth index was created and the computation of wealth quintile, please refer to [19]). Variables representing the socio-economic condition of the community were created as the proportions, respectively, of total households in the PSUs following the Muslim religion, belonging to ST/SC groups, and the poorest wealth category.

### Statistical analysis

Descriptive statistics were used to depict the distribution of the sample, hemoglobin levels and BMI levels, across the covariates. We applied individual-level sampling weights to compute descriptive statistics. We fitted a series of five-level random intercept multiple linear regression models to estimate the association of community-level women’s education with adolescent hemoglobin levels and, separately, with BMI. This was an appropriate choice given our interest in the influence of the context on an individual-level outcome [49]. The five hierarchical population levels were adolescent *i*, nested in household *j*, PSU (community) *k*, district *l* and state *m*. The random components at each level were assumed to follow a normal distribution, with a mean of zero, and a constant variance; and assumed to be mutually independent. Alpha was set at 0·05. The unadjusted Model 1 included community-level women’s education as the predictor. We incrementally added individual-level covariates: adolescent’s gender, age and, adolescent’s education in Model 2, and mother’s education in Model 3. The fully adjusted Model 4 additionally included father’s education, household-level covariates: family size, social group, religion, household wealth; the community-level covariates: wealth, and social and religious composition; and the state-level covariate: region. Firstly, we fit unweighted multilevel models. Additionally, in order to account for the complex sampling design, rescaled weights were computed at the individual-level, using two methods [50]. Method A scaled the weights so that the new weights sum to the cluster sample size and method B scaled the weight so that the new weights sum to the effective cluster size [50]. Method A and method B are the nomenclature used by Carle (2009), following Asparouhov’s labels [50]. We ran our fully adjusted five-level models for hemoglobin and body mass index (BMI) applying the scaled weights obtained using both the methods at the individual-level. We have included these findings in S3 File Weighted multilevel models. Statistical analysis was carried out using Stata, version 12·1 [51].

### Ethical concerns

We carried out a secondary data analysis of publicly available deidentified data collected as a part of NFHS-4 India, accessed via the Demographic and Health Surveys (DHS) Program website. Necessary approvals were sought from the DHS Program to use NFHS-4 data for this project. As we were undertaking secondary data analysis of publicly available deidentified data, and were not collecting any new data, our study was exempt from review at the Institutional Ethics Committee of the host institute of the authors. International Institute for Population Sciences (IIPS), Mumbai, was the nodal agency for the NFHS-4 project [19]. The IIPS Institutional Review Board and the ICF Institutional Review Board granted ethical approval to the NFHS-4 project [19]. Several field agencies selected by IIPS, Mumbai, collected the data for NFHS-4 [52]. Specially trained health investigators hired by the field agencies carried out anthropometric measurements and biomarker testing [52]. The health investigators obtained the written informed consent from the parent or guardian or any other responsible adult in the household for unmarried adolescents aged 15 through 17 years, separately for each measurement and biomarker testing [52]. After receiving the informed consent, the health investigators sought the adolescent’s assent for performing the measurements and tests [52].

## Results

### Descriptive statistics

Our analytical sample for the hemoglobin analysis consisted of a total of 62,648 adolescents aged 15 to 17 years (average: 15.95; SD: 0·81), from 57,638 households, 23,448 PSUs, 640 districts, and 36 states/UT (Table 1). Average hemoglobin value for girls was 11·72 (SD 1·57) g/dl which is lower than the normal range (12 through 14 g/dl) for girls and would be considered as anemia. The interquartile range [25th percentile- 75th percentile] of hemoglobin value among girls was [10.8 g/dl, 12.8 g/dl]. On the other hand, for boys, average hemoglobin value was 13·52 (SD 1·69) g/dl which is within the normal range (13 through 15 g/dl) for boys. The interquartile range of hemoglobin value among boys was [12.5 g/dl, 14.7 g/dl].

**Table 1:** The distribution of the adolescents and average hemoglobin levels in gram/deciliter (g/dl) (N = 62,648) by gender and sociodemographic characteristics.

	Girls		Boys		Total n (column %)
	n (column %)	Hemoglobin level Mean (SD)	n (column %)	Hemoglobin level Mean (SD)	
Religion					
Hindu	43705.57(80.59)	11.65(1.52)	6876.71(81.71)	13.46(1.65)	50582.28(80.74)
Muslim	7836.52(14.45)	11.72(1.46)	1148.78(13.65)	13.49(1.59)	8985.3(14.34)
Christian	1073.79(1.98)	11.81(2.95)	158.22(1.88)	13.62(2.94)	1232.01(1.97)
Other	1616.11(2.98)	11.69(1.8)	232.28(2.76)	13.31(2.08)	1848.39(2.95)
Caste background					
Scheduled Caste	12202.2(22.5)	11.55(1.53)	1855.73(22.05)	13.36(1.66)	14057.93(22.44)
Scheduled Tribe	5168.31(9.53)	11.53(2.1)	754.07(8.96)	13.2(2.29)	5922.38(9.45)
Other Backward Caste	25201.61(46.47)	11.69(1.48)	3812.45(45.3)	13.52(1.63)	29014.06(46.31)
General	11659.88(21.5)	11.79(1.43)	1993.75(23.69)	13.56(1.55)	13653.63(21.79)
Wealth quintile					
Poorest	11719.54(21.61)	11.55(1.58)	1488.79(17.69)	13.14(1.72)	13208.33(21.09)
Poorer	12131.7(22.37)	11.63(1.61)	1790.92(21.28)	13.37(1.73)	13922.62(22.23)
Middle	11318.22(20.87)	11.65(1.6)	1726.96(20.52)	13.43(1.7)	13045.18(20.82)
Richer	10255.27(18.91)	11.72(1.51)	1734.54(20.61)	13.5(1.66)	11989.81(19.14)
Richest	8801.85(16.23)	11.81(1.42)	1673.94(19.89)	13.86(1.57)	10475.79(16.72)
Place of residence					
Urban	16443.14(30.32)	11.72(1.45)	2880.8(34.23)	13.64(1.54)	19323.94(30.85)
Rural	37788.86(69.68)	11.64(1.6)	5535.2(65.77)	13.37(1.76)	43324.06(69.15)
Region					
North	7348.44 (13.55)	11.7(1.91)	1290.17(15.33)	13.62(1.98)	8638.61(13.79)
Central	16931.23 (31.22)	11.69(1.62)	2242.86(26.65)	13.34(1.76)	19174.09(30.61)
East	11920.19 (21.98)	11.48 (1.33)	1616.71(19.21)	13.22 (1.48)	13536.9(21.61)
Northeast	1464.26 (2.7)	12.06 (3.16)	226.39 (2.69)	13.77 (3.11)	1690.65(2.7)
West	7196.59 (13.27)	11.79 (1.11)	1477.01(17.55)	13.47 (1.27)	8673.6(13.85)
South	9371.29 (17.28)	11.65 (1.28)	1562.01(18.56)	13.72(1.4)	10933.3(17.45)
Mother’s education					
Below tenth-grade	45896.54(84.63)	11.63(1.59)	7061.87(83.91)	13.4(1.7)	52958.41(84.53)
Tenth-grade or above	8335.46(15.37)	11.86(1.37)	1354.13(16.09)	13.79(1.59)	9689.59(15.47)
Father’s education					
Below tenth-grade	38423.37(70.85)	11.63(1.58)	5881.94(69.89)	13.39(1.69)	52958.41(84.53)
Tenth-grade or above	15808.63(29.15)	11.76(1.49)	2534.06(30.11)	13.79(1.59)	18342.69(29.28)
Total	54232(86.57)	11.72(1.57)	8416(13.43)	13.52(1·69)	62648(100)

(As we have used weighted frequencies and proportions, the cells contain values up to two decimal points, and the sum of the sample sizes in the subcategories of several socio-demographic characteristics do not exactly match the total.)

The analytical sample for the BMI analysis consisted of a total of 62,846 adolescents aged 15 to 17 years (average: 15.95; SD: 0·81), from 57,813 households, 23,499 PSUs, 640 districts, and 36 states/UT (Table 2). Average BMI values for girls was 19.21 (SD 2.88) kg/m2 and for boys was 19 (SD 3.12) kg/m2 which were within the normal range (18.5 through 23 kg/m2) for both girls and boys, with girls having higher average values than boys. The interquartile range of BMI value among girls was [17.34 kg/m2, 20.64 kg/m2] and the same among the boys was [16.94 kg/m2, 20.44 kg/m2]. Further, an economic gradient was observed in average BMI values among both boys and girls. The average BMI values were lowest among the poorest quintiles. The average BMI values were higher at higher levels of the wealth index, with the richest having the highest average BMI values among both boys and girls. While average BMI values for the girls were within the normal range across all the wealth quintiles, the trend in the interquartile ranges of BMI for girls across the wealth quintiles suggested an inverse association of BMI with wealth (S1 File, Wealth index wise interquartile range of BMI). Fitting this pattern, the average BMI values were lower than normal among the boys in the poorest and poorer categories. The samples comprised mainly of girls (about 87%). The distribution of adolescents by background characteristics in our samples were comparable with that of the Indian adolescent population (Census 2011) [53].

**Table 2:** The distribution of the adolescents and average body mass index in kilogram/squared meter (kg/m^2^) (N = 62,846) by gender and sociodemographic characteristics.

	Girls		Boys		Total n (column %)
	n (column %)	BMI Mean (SD)	n (column %)	BMI Mean (SD)	
Religion					
Hindu	43783.75 (80.51)	19.06 (2.83)	6913.42(81.69)	18.88(3.08)	50697.17 (80.68)
Muslim	7890.97 (14.51)	19.25 (2.97)	1158.58(13.69)	18.91(3.22)	9049.55 (14.4)
Christian	1087.66 (2.00)	20.02 (5.43)	158.26 (1.87)	19.71(6.35)	1245.92 (1.98)
Other	1615.18 (2.97)	19.16 (3.33)	232.73(2.75)	19.32(4.36)	1847.91 (2.94)
Caste background					
Scheduled Caste	12214.42 (22.46)	18.96 (2.66)	1866.94(22.06)	18.76(2.78)	14081.36(22.41)
Scheduled Tribe	5171.82 (9.51)	18.96(3.54)	756.59 (8.94)	18.83(3.6)	5928.41 (9.43)
Other Backward Caste	25293.53 (46.51)	19.05(2.76)	3832.89(45.29)	18.78(3.12)	29126.42 (46.35)
General	11703.22 (21.52)	19.48 (3.15)	2006.58(23.71)	19.34(3.37)	13709.8 (21.81)
Wealth quintile					
Poorest	11681.47 (21.48)	18.69 (2.4)	1492.87(17.64)	18.15(2.53)	13174.34 (20.96)
Poorer	12111.09 (22.27)	18.8 (2.67)	1793.31(21.19)	18.43(2.7)	13904.4 (22.12)
Middle	11355.17 (18.99)	18.97 (2.88)	1734.07(20.49)	18.83(3.32)	13089.24 (20.83)
Richer	10327.33 (18.99)	19.31 (2.99)	1746.76(20.64)	19(3.02)	12074.09(19.21)
Richest	8907.94 (16.38)	20.04 (3.51)	1695.14(20.03)	20.08(3.73)	10603.08 (16.87)
Place of residence					
Urban	16592.25(30.51)	19.6 (3.23)	2911.27(34.4)	19.49(3.31)	19503.52 (31.03)
Rural	37790.75 (69.49)	18.9 (2.74)	5551.73(65.6)	18.61(3.06)	43342.48 (68.97)
Region					
North	7385.21 (13.58)	19.17 (3.4)	1295.69(15.31)	19.19(3.63)	8680.9 (13.81)
Central	16934.87 (31.14)	19 (2.73)	2252.85(26.62)	18.48(3.18)	19187.72 (30.53)
East	11871.81(21.83)	18.98 (2.53)	1622.36(19.17)	18.68(2.61)	13494.17 (21.47)
Northeast	1484.66 (2.73)	19.74 (6.12)	233.58 (2.76)	19.8(6.31)	1718.24 (2.73)
West	7232.94 (13.3)	18.99 (2.55)	1476.79 (17.45	19.8(2.78)	8709.73 (13.86)
South	9473.52 (17.42)	19.42 (2.55)	1582.86(18.69)	19.27(2.68)	11056.38 (17.59)
Mother’s education					
Below tenth-grade	45959.07(84.51)	18.98(2.83)	7086.92(83.74)	18.66(2.99)	53045.99(84.41)
Tenth-grade or above	8423.93(15.49)	19.85(3.3)	1376.08(16.26)	20.2(3.88)	9800.01(15.59)
Father’s education					
Below tenth-grade	38448.78(70.7)	18.96(2.81)	5900.4(69.72)	18.65(2.99)	44349.18(70.57)
Tenth-grade or above	15934.22(29.3)	19.48(3.19)	2562.6(30.28)	19.52(3.57)	9800.01(15.59)
Total	54383 (86·53)	19.21 (2.88)	8463 (13·47)	19.00(3.12)	62846 (100·00)

(As we have used weighted frequencies and proportions the cells contain values up to two decimal points and the sum of the sample sizes in the subcategories of several socio-demographic characteristics do not exactly match the total.)

**Fig 1:**
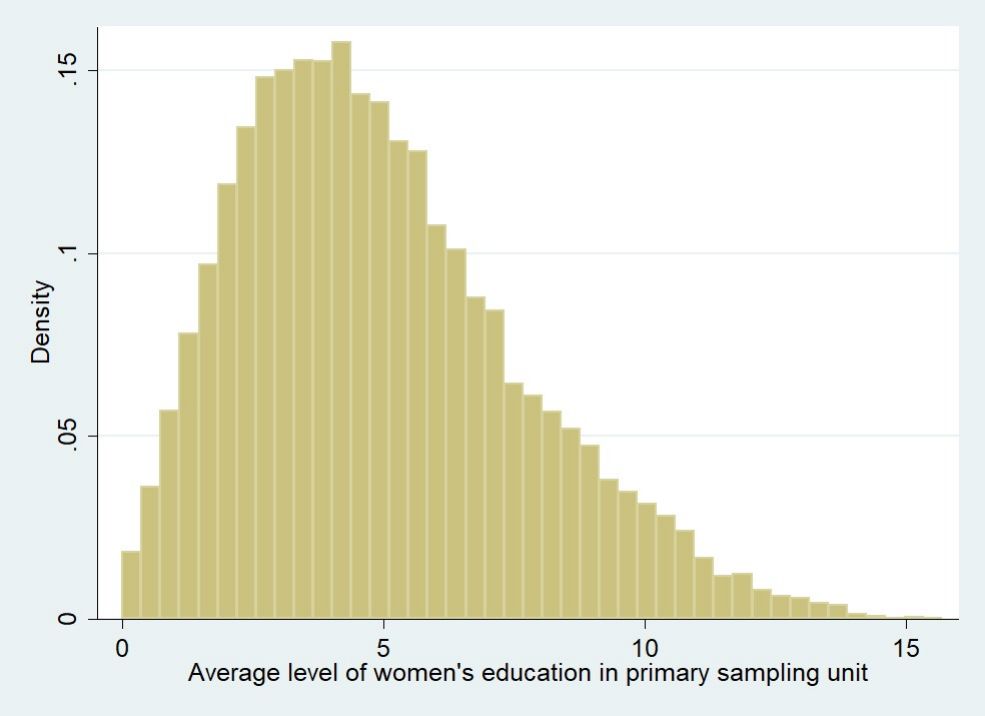
Frequency distribution of primary sampling units by average level of women (above 19 years of age)’s education in completed years of schooling. (n = 23499) (Graph prepared using Stata 12 [54]).

The community-level descriptive statistics were identical for the hemoglobin and BMI analyses, while the sample sizes were 23,448 for the hemoglobin analysis and 23499 for the BMI analysis (Table 3). The proportion of women with formal education was 73.73% in the urban areas as compared to 49.93% in the rural areas. The average (SD) proportion of women having a tenth-grade education in urban PSUs was 0.40 (0.21) whereas the same for rural PSUs was 0.17 (0.13). We have plotted the average value of women’s education (as the number of completed years of schooling) at community (PSU)-level using a histogram (Fig 1). The average number of completed years of schooling among women at the community-level was between four to five years in the maximum number of communities. The average education level among women (in the number of completed years of schooling) at the state- and regional-levels are provided in the S2 File (Women’s education at State and Region levels).

**Table 3:** Summary measures of community-level factors (N = 23,448 for hemoglobin analysis/23499 for BMI analysis)*.

Characteristic	Mean (SD)	25th percentile	75th percentile
Proportion of women having completed formal education at least until 10th grade	0·24 (0·19)	0·09	0·33
Proportion of households belonging to SC/ST group	0·37 (0·32)	0·10	0·57
Proportion of households following Muslim religion	0·11 (0·24)	0·00	0·09
Proportion of households in the poorest wealth category	0·23 (0·27)	0·00	0·38

*Community-level summary statistics were identical for both hemoglobin and BMI analysis.

### Hemoglobin analysis

In the bivariate Model 1 (Table 4), a one standard deviation (0·19) higher community-level women’s education was associated with (0·569(*β*)*0·19) 0·11 g/dl higher hemoglobin level, on average (p<0·001). Adding individual level covariates reduced the value of the coefficient (*β* = 0·423 in Model 2), but retained the positive association of the primary predictor observed in Model 1 (p<0·001). Adding mother’s education (Model 3) further reduced the *β* (0·286), but retained the positive association between the primary predictor and the outcome (p<0·001). The average hemoglobin level in adolescents was higher by 0·045 g/dl if the proportion of women who were educated up to the tenth-grade or above in a community was 19 percentage points higher (p<0·001) in the fully adjusted model (Model 4).

**Table 4:** The association (regression coefficients and 95% CI) of community-level women’s education with adolescent hemoglobin level in unadjusted and adjusted models (N in all models = 62,648 adolescents).

Characteristics	Model 1	Model 2	Model 3	Model 4
Fixed part	Coefficient (95% confidence interval)			
Community-level women’s education	0·569 (0·477, 0·661)***	0·423(0·335, 0·510)***	0·286 (0·191, 0·381)***	0·234 (0·117, 0·352)***
Adolescent’s age		0·010 (-0·005, 0·025)	0·014 (-0·001, 0·028)	0·013 (-0·002, 0·027)
Adolescent’s female gender (Reference category: boys)		-1·791(-1·826, -1·756)***	-1·790 (-1·826, -1·755)***	-1·791 (-1·826, -1·755)***
Adolescent’s education		0·018 (0·013, 0·024)***	0·015 (0·009, 0·020)***	0·013 (0·007, 0·018)***
Mother’s education			0·012 (0·009, 0·015)***	0·007 (0·003, 0·011)***
Father’s education				0·003 (-0·001, 0·006)
Family size				-0·001 (-0·006, 0·004)
Social group (reference category: Scheduled Caste)				
Scheduled Tribe				-0·103 (-0·155, -0·051)***
Other backward classes				0·089 (0·051, 0·127)***
General				0·081 (0·036, 0·126)***
Religion (reference category: Hindu)				
Muslim				0·057 (-0·001, 0·116)
Christian				0.125 (0·036, 0·215)**
Other				-0·036 (-0·114, 0·042)
Household wealth (reference category: poorest)				
Poorer				-0·004 (-0·045, 0·037)
Middle				0·023 (-0·024, 0·070)
Richer				0·011 (-0·043, 0·065)
Richest				0·095 (0·031, 0·160)**
Proportion of SC and ST households				-0·045 (-0·115, 0·025)
Proportion of Muslim households				0·080 (-0·008, 0·168)
Proportion of poorest households				0·031 (-0·010, 0·071)
Place of residence (reference category: Urban)				
Rural				0·031 (-0·010, 0·071)
Region (reference category: North)				
Central				0.207 (-0·198, 0·611)
East				-0·062 (-0·433, 0·308)
Northeast				0·740 (0·422, 1·059)***
West				0·142 (-0·246, 0·529)
South				-0·011 (-0·332, 0·310)
Constant	11·865 (11·719, 12·012)***	13·12 (12·846, 13·394)***	13·075 (12·802, 13·349)***	12·867 (12·536, 13·198)***
Random part	Variance (95% confidence interval)			
State	0·171 (0·098, 0·298)	0·174 (0·101, 0·300)	0·170 (0·099, 0·293)	0·085 (0·046, 0·156)
District	0·127 (0·110, 0·147)	0·125 (0·108, 0·144)	0·124 (0·107, 0·143)	0·119 (0·103, 0·138)
Primary sampling unit	0·197 (0·176, 0·221)	0·145 (0·126, 0·166)	0·144 (0·126, 0·165)	0·141 (0·123, 0·162)
Household	0·435 (0·374, 0·506)	0·429 (0·374, 0·492)	0·428 (0·373, 0·491)	0·428 (0·373, 0·490)
Residual	2·034 (1·970, 2·100)	1·724 (1·667, 1·782)	1·724 (1·667, 1·782)	1·722 (1·665, 1·780)

*p<0.05,

**p<0.01,

***p<0.001.

### BMI analysis

Similar to the relationship of contextual education with hemoglobin, a higher proportion of women educated at least until the tenth-grade in the community was associated with higher adolescent BMI. The unadjusted Model 1 for BMI analysis estimated that one standard deviation higher community-level women’s education was associated with ((*β*) 1.911*0.19) 0.36 kg/m^2^ higher average BMI (p<0.001), which was reduced in the fully adjusted Model 4 to (0.776(*β*)*0.19) 0.15 kg/m^2^ (p<0.001). The positive relationship of the main predictor with the outcome was sustained across all the models (p<0.001). Additionally, Model 4 for BMI analysis suggested that higher proportions of poorest or Muslim households in a community were significantly positively associated with BMI. Table 5.

**Table 5:** The association (regression coefficients and 95% CI) of community-level women’s education with adolescent BMI in unadjusted and adjusted models (N in all models = 62,846 adolescents).

Characteristics	Model 1	Model 2	Model 3	Model 4
Fixed part	Coefficient (95% confidence interval)			
Community-level women’s education	1.911(1.761, 2.062)***	1.789(1.634, 1.944)***	1.353(1.183, 1.524)***	0.776(0.567, 0.985)***
Adolescent’s age		0.300(0.273, 0.327)***	0.312(0.285, 0.339)***	0.308(0.281, 0.334)***
Adolescent’s female gender (Reference category: boys)		0.261(0.196, 0.326)***	0.263(0.198, 0.328)***	0.272(0.207, 0.337)***
Adolescent’s education		0.024 (0.015, 0.034)***	0.012 (0.002, 0.021)*	0.006 (-0.005, 0.016)
Mother’s education			0.038 (0.031, 0.044)***	0.013 (0.005, 0.020)**
Father’s education				0.004(-0.003, 0.010)
Family size				-0.023 (-0.033, -0.013)***
Social group (reference category: Scheduled Caste)				
Scheduled Tribe				0.119 (0.025, 0.213)*
Other backward classes				-0.019 (-0.089, 0.051)
General				0.171 (0.088, 0.254)***
Religion (reference category: Hindu)				
Muslim				0.015 (-0.095, 0.124)
Christian				0.330 (0.175, 0.486)***
Other				0.180 (0.039, 0.320)*
Household wealth (reference category: poorest)				
Poorer				0.074 (-0.002, 0.150)
Middle				0.205 (0.118, 0.292)***
Richer				0.334 (0.234, 0.433)***
Richest				0.851 (0.733, 0.969)***
Proportion of SC and ST households				0.047 (-0.077, 0.170)
Proportion of Muslim households				0.168 (0.011, 0.326)*
Proportion of poorest households				0.227 (0.067, 0.387)**
Place of residence (reference category: Urban)				
Rural				-0.133 (-0.205, -0.062)***
Region (reference category: North)				
Central				-0.101 (-0.560, 0.357)
East				0.003 (-0.418, 0.423)
Northeast				1.010 (0.734, 1.465)***
West				-0.0386 (-0.479, 0.402)
South				0.185 (-0.180, 0.550)
Constant	19.085 (18.866, 19.30)***	13.888 (13.409, 14.367)***	13.747 (13.269, 14.224)***	13.534 (13.020, 14.048)***
Random part	Variance (95% confidence Intervals)			
State	0.400 (0.244, 0.657)	0.406 (0.248, 0.666)	0.392 (0.240, 0.643)	0.109 (0.060, 0.198)
District	0.087 (0.068, 0.112)	0.086 (0.067, 0.110)	0.086 (0.067, 0.110)	0.077 (0.059, 0.100)
Primary sampling unit	0.326 (0.264, 0.402)	0.319 (0.258, 0.395)	0.313 (0.252, 0.389)	0.288 (0.228, 0.363)
Household	2.126 (1.928, 2.344)	2.239 (2.044, 2.454)	2.232 (2.037, 2.446)	2.214 (2.020, 2.427)
Residual	5.559 (5.365, 5.760)	5.381 (5.191, 5.577)	5.376 (5.186, 5.572)	5.365 (5.176, 5.560)

*p<0.05,

**p<0.01,

***p<0.001.

Notably, nearly 28% of the PSUs in our sample had less than 10% of the women with a tenth-grade education. If 100% of the women in these communities acquire at least a tenth-grade education, our findings suggest that it might lead to (0·9*0·234(*β*)) 0·21 g/dl higher hemoglobin level, and (0·9*0·776(*β*)) 0·7 kg/m^2^ higher BMI, on average, *among all adolescents* in those communities. Similarly, in the nearly 52% of the PSUs having less than 20% of the women with a tenth-grade education, the estimated gain in hemoglobin level would be 0·19 g/dl and in BMI would be 0·62 kg/m^2^, on average, for *all adolescents* in such communities.

In all the models, the unexplained variance at all the five levels remained statistically significant, indicating the importance of contextual factors at the household-, community-, district-, and state-levels on adolescent hemoglobin level and BMI.

## Discussion

Our study of more than 62,000 Indian adolescents aged 15 through 17 years found that community-level women’s education was positively associated with their hemoglobin level and BMI, after accounting for relevant covariates and contextual levels. Further, we report evidence of the importance of the context in general on adolescent hemoglobin and BMI. To our knowledge, this is the first attempt to examine the influence of contextual factors on adolescent health or nutritional status. These findings are especially relevant considering that 52% of communities in a large community-based sample from India had only 20% or fewer women with a tenth-grade education.

We acknowledge that while we have focused on the larger issue of undernutrition among adolescents, overweight is a growing concern, albeit not yet widely prevalent, among Indian adolescents. Nearly 5% of the adolescents aged 10 to 19 years are reported to be overweight or obese [5]. It is likely that following a widely documented pattern observed in several countries undergoing nutritional transition, we might observe a positive association of maternal education with an adolescent’s BMI. However, whether living in a community that values women is likely to increase the risk of overweight in adolescents remains to be investigated. Our goal was the focus on the widely prevalent and persistent issue of undernutrition among adolescents that, in our view, requires sustained efforts. Given the current levels, and recent trajectories, of nutrition-sensitive and -specific determinants in India, and the upheaval caused by the pandemic, we argue that undernutrition requires even more attention than “business as usual”.

Our findings are comparable with a few previous studies. Haverkate et al. [55] reported that the average years of education in adults at the community-level was positively associated with hemoglobin level among reproductive age women across 21 African countries (*β* 0·290g/l, p<0·05). Although their use of education-level was different from our conceptualization, their finding is similar to ours in terms of the direction of the relationship. Our findings are also similar to those of the previous studies that examined the relationship of contextual determinants with child health outcomes in the Indian context. Kravdal’s 2004 study [39] of 90000 Indian mothers of under-fives, from a nationally representative survey, demonstrated that the average years of education among women at the community-level was associated with lower child mortality (*β* -0·045, p<0·01) independent of the child’s mother’s education. Using 1994 data from 5,623 infants living in 195 districts of 16 states Parashar [31] suggested that the average female literacy rate at the district-level was associated with higher complete immunization status of children under-two, independent of their mother’s education (*β* 1·49, p<0·05). Burroway [37] in 2016 showed that the percentage of female secondary school enrolment at the country-level reduced the odds of stunting in under-fives (*β* 0·991, p<0·05) across 50 developing countries. Although the size of the estimates for community-level women’s education was smaller in our study as compared to these previous studies, notably, its association with the outcome was in the expected direction. Furthermore, the coefficient’s estimate is not strictly comparable across the studies, as the operationalization of community-level women’s education, the outcome of interest, the study population, and the nature of causal influence were different in all the studies. Moreover, adolescent health might be less influenced by community-level women’s education than the health of under-fives: adolescents might get affected by a greater number of determinants, so the change in the outcome influenced by one particular determinant might be smaller in comparison. However, as our findings are applicable to a large number of adolescents, even a small change in hemoglobin level or BMI of adolescents due to the influence of the context has a large effect at the population level.

Our findings are in line with recent evidence [56] from rural Odisha, positing social and unequal gender norms in community that deprioritize women’s preventive health, especially in non-pregnant women of reproductive age, as important beyond individual-level factors, and resulting in poor uptake of iron supplementation. Although the focus of their qualitative inquiry was on exploring the barriers to iron supplementation in women [56], they have identified the need for addressing social norms as extra-individual level factors in reducing the anemia burden in India. Triangulating our quantitative evidence, derived from a nationally representative survey with the qualitative understanding of barriers to anemia prevention from rural Odisha [56], adds strength to our argument, that socio-cultural norms valuing women’s well-being, as captured by community-level women’s education, are an important determinant of adolescent hemoglobin.

Our investigation employed a measure of women in India completing education up to the tenth-grade, which itself is a bar set too low. Completing high-school education requires completing education till the twelfth-grade. We believe that ideally, schooling up to twelfth grade should be encouraged among women (and men) as the basic minimum; notably, it is prioritized in the National Education Policy 2020 [26]. However, our selection of a tenth-grade education as a cut-off serves two inter-related purposes: first, it captures the gender disparity in education more sharply than twelfth-grade schooling (in our dataset the gender disparity is 14.38% at the tenth grade as compared to 9.23% in the twelfth grade [19]; this might be so because the percentage of the population that has completed the twelfth-grade would have been low for both boys and girls combined, at least in the earlier times completing the twelfth grade was not historically that common), and second, it is the minimum grade at which the rigor of assessment of learning is somewhat reliable, at least after the introduction of RTE Act 2009.

Our findings suggest that if at least a tenth-grade education is ensured among all women, it will potentially lead to appreciable gains in adolescent nutrition. Thus, ensuring the education of girls beyond the tenth-grade might have an even greater influence on adolescent hemoglobin and BMI. However, our core interest is in moving beyond increasing the number of years of completed schooling among women and directing attention to the socio-cultural norms in a community that values women and facilitates their completing high school (or more). We argue that community-level women’s education is a crude proxy for the strength of socio-cultural norms of a community that values women. Further, measuring it as the number of completed years of schooling fails to capture the quality of the education acquired, which is frequently of poor quality and inequitably distributed in India [57]. Therefore, it is a weak proxy for capturing the health-promoting impacts of schooling, such as an enhanced critical thinking ability, empowerment conducive towards healthy behaviors, and a change in social norms. It is reported that the curriculum in Indian schools lacks an emphasis on engaging children and inspiring critical thinking among them [58]. Despite the use of such a crude indicator, we were able to find modest statistical evidence supporting our hypothesis. Moreover, by including the mother’s education as a covariate, one of the important mediators through which community-level women’s education likely impacts adolescent health, we present a conservative estimate of the potential impact of community-level women’s education on adolescent nutrition. Thus, despite the coefficient being small, it gives a valuable insight about the influence on adolescent nutrition of community-level women’s education and all that this indicator represents.

Our findings indicated that living in communities having a higher proportion of poor households was associated with a higher BMI in adolescents, after accounting for the other covariates. We suspect that this counter-intuitive association might indicate that communities with a higher proportion of poor households might have a lower cost of living in comparison to communities with a lower proportion of poor households. This might be associated with relatively higher proportion of household income being available to be spent of food and health, which in turn result in higher adolescent BMI. The communities with a higher proportion of Muslim households might be suggestive of the predominance in an area of households with non-vegetarian food habits which in turn would be associated with higher BMI in these communities.

We have not empirically examined the potential pathways through which the relationship of community-level women’s education with adolescent undernutrition operates. However, theoretically, a contextual impact [32] is plausible as theorized in the case of child health [37, 39, 59], which, by extension, might be relevant for adolescents. Adolescents living in a neighborhood might benefit from a contextual effect of having a large proportion of educated women in the neighborhood who, leveraging their education, may have a greater access to information and a widened social network, for instance through working outside their home, that exposes them to new knowledge and behavior norms such as those related to (adolescent’s) nutrition, and education [60]. Knowledge and behaviors of educated women who are more likely to break from traditions in adopting advances in nutrition [37], and might be better equipped to use available resources to improve dietary quality [61] and nutritional status [10] of their children. For instance, they might be more aware about dietary requirements and sources of nutrients including iron, and ensure its regular consumption among adolescent children [62]. Educated women might be prompt in using health services for children and able to navigate health systems better [37, 59, 62]. Education might enhance women’s participation in household decision making, increasing her bargaining power to divert resources towards nutrition, health and education of her children [37]. Favorable attitudes and progressive behaviors towards adolescent health among a substantially higher number of educated women in the neighborhood likely *exposes other community members to the health-promoting choices made*, *and advocated*, *by educated women*. Through processes such as *social learning and social influence* [63] *it likely spreads to other community members including the adolescents and their mothers*. Perhaps this *alters social norms and expectations* related to adolescent care, nutrition, health and education. Further, a critical number of educated women in the community may *generate the social capital needed to change a community’s ability* to better use the available health services and make local health workers more responsive to the demands of the community [59]. Educated women might also *contribute towards expansion of economic wealth creation in the community*, leading to investments in health, sanitation and education [39]. Thus, altered normative expectations and community wealth creation by educated women *do not simply benefit their offspring but might also benefit adolescents in the community*.

While our focus was on the immediate neighborhood of residence as a community, (village/village cluster/urban wards) we also found evidence of the influence on adolescent hemoglobin of the context at state- and district-levels, even after accounting for a host of covariates. These findings underscore the potential relevance of context on adolescent health.

This indicates that it is unrealistic to expect major improvement in adolescent health through attempts focused exclusively on attributes of the adolescents without modifying the structural level determinants at higher levels.

India’s key intervention to reduce anemia burden- *Anemia Mukt Bharat* noticeably prioritizes maximizing iron supplementation [64]. Although essential, iron supplementation might be effective in reducing anemia to a limited extent, and in certain contexts [65]. Notably, less than half of the anemia in developing countries is attributable to iron deficiency [66]. This might explain the ineffectiveness of India’s long history of iron supplementation in reducing anemia. Several other biological factors, such as poor bioavailability of iron, poor iron absorption, deficiency of other nutrients, exposure to recurrent infections and parasitic infestations, inadequate iron stores, and inadequate replacement of lost iron during menstruation in girls are involved in causing anemia [67]. Programs such as Rashtriya Kishor Swasthya Karyakram, the Mid-day meal program (school-based supplementary nutrition program) and POSHAN Abhiyaan include strategies to improve adolescent nutrition. While the coverage of these programs is low and variable across states [5], their primary focus is on providing supplementary nutrition to the adolescents with a relatively little focus on addressing the contextual social determinants of adolescent nutrition.

Furthermore, behavior change communication carried out through schools [68] and peer educators [69] among adolescents is targeted at the individual-level. Adolescence is a transitional phase in which increasing autonomy and time spent outside the home increase the potential influence of peers and others outside the family on adolescent’s behaviors [70]. On the other hand, adolescents have a limited opportunity to participate in decisions related to household purchases of food items [5]. While creating awareness related to health and nutrition in adolescents is crucial, we argue that an individual-level approach is insufficient to realize effective behavior change in this backdrop. The poor uptake of iron supplementation in urban girls despite efforts to increase awareness [71], and motivation from parents, teachers and friends increasing its uptake in girls on the other hand [72] suggests the same. We argue that we need to consider adolescents as social beings embedded in their communities [9], who need support from the family and community to engage in health-enhancing behaviors. Behavior change efforts need to emphasize modifying the local context within which adolescents and their families make decisions. Our findings suggest that raising the level of women’s education could be an important factor facilitating this process.

Summing up, drawing from the Ecosocial theory [9], undernutrition in adolescents may be conceptualized as an embodiment of poor living conditions and disadvantaged social positions. It is imperative that adolescent health related policy actions are sensitive in addressing the contextual determinants of adolescent health and advocate for structural changes. Evidence suggests that investing in women’s education is among the best investments that could facilitate economic development, socio-political transformations, and improved health of generations [73]. We argue that such an investment is not just the means to an end (better nutrition of adolescents) but also an important outcome itself, which ideally reflects the greater value placed on women’s well-being in general. There is an evident gender gap in literacy rates and enrolment ratios at every level of formal education in India; and is more pronounced among the socio-economically marginalized women [26]. Our findings advocate for the strengthening of policies encouraging women’s education, with a special focus on socio-economically disadvantaged women, at every level of formal education. In India, nearly 40% of girls aged 15 to 18 years are out of school [74]. Discriminatory gender norms valuing and prioritizing a boy’s education over a girl’s, poverty, early marriage and child bearing, viewing girls as liabilities, dowry practices, child labor, household work, and care responsibilities are important demand-side barriers to girls’ education [58]. On the other hand, an evaluation of the supply side points towards an insufficiently funded education system, with severe shortage of secondary schools and teachers especially in rural areas [74]. Not having a school nearby increases the opportunity cost of sending girls to school, which puts poor girls at double disadvantage: because of gender and poverty. Concerns over girls’ safety and security are also an important barrier in girls’ education [75]. Thus, women’s education is a consequence of structural level factors, social norms and attitude towards women’s education, parental attitudes and household level factors that collectively determine availability and access to education for women. This therefore points towards an urgent need for a convergent action, directed towards structural and socio-cultural transformations that support and facilitate women’s education beyond the elementary level.

Our study has several limitations. We included a narrow age-range of adolescents in our analysis because of the data constraints. Research suggests that early (10 to 14) and late (15–19) adolescents differ in their health status and its determinants [5]. It is possible that younger adolescents (10 to 14 years), who likely have less autonomy than the older adolescents, could be more affected by several aspects related to the norms of care, feeding practices, and other health behaviors in the family; which in turn are arguably affected by the broader socio-cultural norms and behaviors of the educated women in the community. We therefore submit that our findings represent a conservative estimate of the impact of community-level women’s education on adolescent health. Further, because of the cross-sectional nature of the data, the association does not imply any directionality or causal relationships. If the association is presumed to be causal, why and how remains unaddressed. The relative importance of the different processes and pathways that we theorize, as well as the role of other contextual determinants, remain to be empirically tested. Next, our analytical sample mainly consisted of unmarried girls (85%), living with both the parents in the same household. Only 15% of the total households selected for the survey were eligible for measuring hemoglobin level and anthropometry in adolescent boys as per the survey design [19]. This limited the representation of boys in our sample. As we aimed to adjust for parental education, we excluded married adolescents and those adolescents whose father did not stay in the same household because information on parent’s education was not available for these adolescents. Our findings therefore are not generalizable to married adolescents or those who are not staying with both their parents. We however surmise that community-level women’s education might have a greater influence on the health of this subgroup of adolescents, than what we found in our sample. Lastly, while large-scale national-level studies could hint towards a broader picture and a general pattern, given that local contextual-level factors affect their health, future research needs to emphasize gaining deeper insight into how adolescents as a diverse group may have unique health needs. This requires a detailed enquiry based on a sound theoretical premise.

The strength and novelty of our findings lie in demonstrating the contextual influences at various population levels on adolescent hemoglobin and BMI while providing policy-relevant evidence of the association of a contextual factor such as community-level women’s education with adolescent undernutrition in the Indian context.

We conclude by reiterating that although the role of social determinants at multiple contextual levels in adolescent health and nutrition is emphasized in literature [70], policy action aiming at adolescent health in India is directed at individual-level biological determinants. Our findings bring the role of contextual factors to the center, highlighting the importance of structural changes and the need to engage communities along with adolescents. This might serve to fill a crucial link in our efforts to improve adolescent health and nutrition. Our findings might also be of relevance for adolescents in similar resource-poor societies that are patriarchal.

## Supporting information

S1 File Wealth index wise interquartile range of BMI. (DOCX)

S2 File Women’s education at State and Region levels in India. (DOCX)

S3 File Weighted multilevel models. (DOCX)

S1 Fig Quantiles of average education level among women in number of completed years of schooling at the state-level according to National Family Health Survey 2015–16. (TIF)

S2 Fig Region-wise average education level among women in number of completed years of schooling according to National Family Health Survey 2015–16. (TIF)
