# Supplementary material for: Community-level women’s education and undernutrition among Indian adolescents: A multilevel analysis of a national survey
Source: PLoS One. 2021 May 20;16(5):e0251427. doi: 10.1371/journal.pone.0251427 (PMC8136857; doi:10.1371/journal.pone.0251427)
Supplement: S1 File — (DOCX) [file pone.0251427.s001.docx]

S1 File. Wealth index wise interquartile range of BMI

S1 Table. Interquartile range for body mass index in kilogram/squared meter (kg/m^2^) stratified by gender and wealth index quintiles (N=62846)

| Wealth index | Girls | | Boys | |
| --- | --- | --- | --- | --- |
|  | 25th percentile | 75th percentile | 25th percentile | 75th percentile |
| Poorest | 17.25 | 20.09 | 16.61 | 19.6 |
| Poorer | 17.29 | 20.4 | 16.94 | 20.03 |
| Middle | 17.3 | 20.72 | 16.84 | 20.35 |
| Richer | 17.37 | 20.92 | 17.07 | 20.73 |
| Richest | 17.64 | 21.59 | 17.39 | 21.79 |
