# Supplementary material for: Community-level women’s education and undernutrition among Indian adolescents: A multilevel analysis of a national survey
Source: PLoS One. 2021 May 20;16(5):e0251427. doi: 10.1371/journal.pone.0251427 (PMC8136857; doi:10.1371/journal.pone.0251427)
Supplement: S2 File — (DOCX) [file pone.0251427.s002.docx]

**S2 File. Women’s education at State and Region levels in India**

S1 Table: Average education level among women in number of completed years of schooling at the state-level according to National Family Health Survey 2015-16.

| **No.** | **State** | **Average education level among women in number of completed years of schooling** |
| --- | --- | --- |
| 1 | Andaman and Nicobar Islands | 7.046404 |
| 2 | Andhra Pradesh | 4.508258 |
| 3 | Arunachal Pradesh | 4.602137 |
| 4 | Assam | 5.422881 |
| 5 | Bihar | 3.140236 |
| 6 | Chandigarh | 9.25701 |
| 7 | Chhattisgarh | 4.581711 |
| 8 | Dadra and Nagar Haveli | 5.457895 |
| 9 | Daman and Diu | 6.226385 |
| 10 | Goa | 7.621145 |
| 11 | Gujarat | 5.115352 |
| 12 | Haryana | 5.920022 |
| 13 | Himachal Pradesh | 6.583068 |
| 14 | Jammu and Kashmir | 4.905863 |
| 15 | Jharkhand | 3.958138 |
| 16 | Karnataka | 5.166354 |
| 17 | Kerala | 9.120028 |
| 18 | Laksadweep | 7.803895 |
| 19 | Madhya Pradesh | 4.050939 |
| 20 | Maharashtra | 5.875762 |
| 21 | Manipur | 6.91273 |
| 22 | Meghalaya | 6.135528 |
| 23 | Mizoram | 6.836338 |
| 24 | Nagaland | 6.062699 |
| 25 | New Delhi | 8.537686 |
| 26 | Odisha | 4.531918 |
| 27 | Puducherry | 8.108173 |
| 28 | Punjab | 6.787355 |
| 29 | Rajasthan | 3.75127 |
| 30 | Sikkim | 6.156322 |
| 31 | Tamilnadu | 6.525507 |
| 32 | Tripura | 5.786355 |
| 33 | Uttar Pradesh | 4.577032 |
| 34 | Uttarakhand | 6.091017 |
| 35 | West Bengal | 4.894469 |
| 36 | Telangana | 4.455838 |
|  | India | 5.10297 |

S2 Table: Average education level among women in number of completed years of schooling across regions according to National Family Health Survey 2015-16.

| **No.** | **Region** | **Average education level among women in number of completed years of schooling** |
| --- | --- | --- |
| 1 | North | 5.47 |
| 2 | Central | 4.4 |
| 3 | East | 3.97 |
| 4 | Northeast | 5.89 |
| 5 | West | 5.63 |
| 6 | South | 6.16 |
|  | India | 5.10297 |
