# Supplementary material for: Community-level women’s education and undernutrition among Indian adolescents: A multilevel analysis of a national survey
Source: PLoS One. 2021 May 20;16(5):e0251427. doi: 10.1371/journal.pone.0251427 (PMC8136857; doi:10.1371/journal.pone.0251427)
Supplement: S3 File — (DOCX) [file pone.0251427.s003.docx]

S3 FILE. Weighted multilevel models

FULLY ADJUSTED FIVE-LEVEL MODELS WITH SAMPLING WEIGHTS

Drawing from Carle (2009), we computed scaled weight using method A which scales the weights so that the new weights sum to the cluster sample size and method B scales the weight so that the new weights sum to the effective cluster size[1]. Method A and method B are the nomenclature used by Carle (2009), following Asparouhov's labels [1]. We ran our fully adjusted five-level models for Hemoglobin and body mass index (BMI) applying scaled weights obtained using both the methods at the individual-level. We also present the findings from the unweighted fully adjusted model, for reference (S1 Table and S2 Table). The value of coefficients do not vary across the three models, however, range of 95% confidence interval varies between the unweighted and the weighted models. We observed a change in p-value between the unweighted and weighted models, however, the findings are statistically significant across both the models (p<0.01). Consistent findings across the unweighted and the weighted models increased our confidence in the results. A caveat is that weights are applied only at the individual-level, and not at all the five levels, which remains a limitation.

**S1 Table: The association (regression coefficients and 95% CI) of community-level women’s education with adolescent hemoglobin level in the fully adjusted models using sampling weights.**

(N in all models = 62,648 adolescents)

| Characteristics | Model 4  Scaled weight  Method A | Model 4  Scaled weight  Method B | Model 4  Unweighted Model |
| --- | --- | --- | --- |
| Fixed part | Coefficient (95% confidence interval) | | |
| Community-level women’s education | 0.234 (0.079,0.39)** | 0.234 (0.079,0.39)** | 0·234 (0·117, 0·352)*** |
| Adolescent’s age | 0.013 (-0.006,0.031) | 0.013 (-0.006,0.031) | 0·013 (-0·002, 0·027) |
| Adolescent’s female gender (Reference category: boys) | -1.791 (-1.861,-1.72)*** | -1.791 (-1.861,-1.72)*** | -1·791 (-1·826, -1·755)*** |
| Adolescent’s education | 0.013 (0.004,0.022)** | 0.013 (0.004,0.022)** | 0·013 (0·007, 0·018)*** |
| Mother’s education | 0.007 (0.003,0.01)*** | 0.007 (0.003,0.01)*** | 0·007 (0·003, 0·011)*** |
| Father’s education | 0.003 (0,0.006) | 0.003 (0,0.006) | 0·003 (-0·001, 0·006) |
| Family size | -0.001 (-0.006,0.004) | -0.001 (-0.006,0.004) | -0·001 (-0·006, 0·004) |
| Social group (reference category: Scheduled Caste) |  |  |  |
| Scheduled Tribe | -0.103 (-0.185,-0.021)* | -0.103 (-0.185,-0.021)* | -0·103 (-0·155, -0·051)*** |
| Other backward classes | 0.089 (0.052,0.127)*** | 0.089 (0.052,0.127)*** | 0·089 (0·051, 0·127)*** |
| General | 0.081 (0.03,0.132)** | 0.081 (0.03,0.132)** | 0·081 (0·036, 0·126)*** |
| Religion (reference category: Hindu) |  |  |  |
| Muslim | 0.057 (-0.011,0.125) | 0.057 (-0.011,0.125) | 0·057 (-0·001, 0·116) |
| Christian | 0.125 (0.033,0.217)** | 0.125 (0.033,0.217)** | 0.125 (0·036, 0·215)** |
| Other | -0.036 (-0.175,0.103) | -0.036 (-0.175,0.103) | -0·036 (-0·114, 0·042) |
| Household wealth (reference category: poorest) |  |  |  |
| Poorer | -0.004 (-0.068,0.061) | -0.004 (-0.068,0.061) | -0·004 (-0·045, 0·037) |
| Middle | 0.023 (-0.054,0.1) | 0.023 (-0.054,0.1) | 0·023 (-0·024, 0·070) |
| Richer | 0.011 (-0.08,0.102) | 0.011 (-0.08,0.102) | 0·011 (-0·043, 0·065) |
| Richest | 0.095** (0.037,0.153) | 0.095**  (0.037,0.153) | 0·095 (0·031, 0·160)** |
| Proportion of SC and ST households | -0.045 (-0.126,0.037) | -0.045 (-0.126,0.037) | -0·045 (-0·115, 0·025) |
| Proportion of Muslim households | 0.08 (-0.009,0.169) | 0.08 (-0.009,0.169) | 0·080 (-0·008, 0·168) |
| Proportion of poorest households | 0.031 (-0.093,0.154) | 0.031 (-0.093,0.154) | 0·031 (-0·010, 0·071) |
| Place of residence  (reference category: Urban) |  |  |  |
| Rural  2.hv025 | 0.031 (-0.016,0.077) | 0.031 (-0.016,0.077) | 0·031 (-0·010, 0·071) |
| Region (reference category: North) |  |  |  |
| Central  region 2 | 0.207 (-0.037,0.45) | 0.207 (-0.037,0.45) | 0.207 (-0·198, 0·611) |
| East  region 3 | -0.062 (-0.311,0.187) | -0.062 (-0.311,0.187) | -0·062 (-0·433, 0·308) |
| Northeast  region 4 | 0.74 (0.361,1.12)*** | 0.74 (0.361,1.12)*** | 0·740 (0·422, 1·059)*** |
| West region 5 | 0.142 (-0.194,0.477) | 0.142 (-0.194,0.477) | 0·142 (-0·246, 0·529) |
| South | -0.011 (-0.304,0.282) | -0.011 (-0.304,0.282) | -0·011 (-0·332, 0·310) |
| Constant | 12.867 (12.512,13.222)*** | 12.867 (12.512,13.222)*** | 12·867 (12·536, 13·198)*** |
| Random part | Variance (95% confidence interval) | | |
| State | 0.085 (0.049,0.145) | 0.085 (0.049,0.145) | 0·085 (0·046, 0·156) |
| District | 0.119 (0.081,0.175) | 0.119 (0.081,0.175) | 0·119 (0·103, 0·138) |
| Primary sampling unit | 0.141 (0.108,0.183) | 0.141(0.108,0.183) | 0·141 (0·123, 0·162) |
| Household | 0.428 (0.375,0.487) | 0.428 (0.375,0.487) | 0·428 (0·373, 0·490) |
| Residual | 1.722 (1.62,1.83) | 1.722 (1.62,1.83) | 1·722 (1·665, 1·780) |

**S2 Table: The association (regression coefficients and 95% CI) of community-level women’s education with adolescent BMI in the fully adjusted models using sampling weights.**

(N in all models = 62,846 adolescents)

| Characteristics | Model 4  Scaled weight  Method A | Model 4  Scaled weight  Method B | Model 4  Unweighted Model |
| --- | --- | --- | --- |
| Fixed part | Coefficient (95% confidence interval) | | |
| Community-level women’s education | 0.776 (0.493,1.059)*** | 0.776 (0.493,1.059)*** | 0.776(0.567, 0.985)*** |
| Adolescent’s age | 0.307 (0.274,0.341)*** | 0.307 (0.274,0.341)*** | 0.308(0.281, 0.334)*** |
| Adolescent’s female gender (Reference category: boys) | 0.272 (0.135,0.409)*** | 0.272 (0.135,0.409)*** | 0.272(0.207, 0.337)*** |
| Adolescent’s education | 0.006 (-0.003,0.015) | 0.006 (-0.003,0.015) | 0.006 (-0.005, 0.016) |
| Mother’s education | 0.013 (0.005,0.02)** | 0.013 (0.005,0.02)** | 0.013 (0.005, 0.020)** |
| Father’s education | 0.004 (-0.002,0.009) | 0.004 (-0.002,0.009) | 0.004(-0.003, 0.010) |
| Family size | -0.023 (-0.031,-0.015)*** | -0.023 (-0.031,-0.015)*** | -0.023 (-0.033, -0.013)*** |
| Social group (reference category: Scheduled Caste) |  |  |  |
| Scheduled Tribe | 0.119 (-0.05,0.288) | 0.119 (-0.05,0.288) | 0.119 (0.025, 0.213)* |
| Other backward classes | -0.019 (-0.089,0.051) | -0.019 (-0.089,0.051) | -0.019 (-0.089, 0.051) |
| General | 0.171 (0.103,0.239)*** | 0.171 (0.103,0.239)*** | 0.171 (0.088, 0.254)*** |
| Religion (reference category: Hindu) |  |  |  |
| Muslim | 0.015 (-0.085,0.114) | 0.015 (-0.085,0.114) | 0.015 (-0.095, 0.124) |
| Christian | 0.33 (0.134,0.527)** | 0.33 (0.134,0.527)** | 0.330 (0.175, 0.486)*** |
| Other | 0.179 (-0.028,0.387) | 0.179 (-0.028,0.387) | 0.180 (0.039, 0.320)* |
| Household wealth (reference category: poorest) |  |  |  |
| Poorer | 0.074 (0.012,0.135)* | 0.205 (0.104,0.306)* | 0.074 (-0.002, 0.150) |
| Middle | 0.205 (0.104,0.306)*** | 0.205 (0.104,0.306)*** | 0.205 (0.118, 0.292)*** |
| Richer | 0.333 (0.21,0.457)*** | 0.333 (0.21,0.457)*** | 0.334 (0.234, 0.433)*** |
| Richest | 0.851 (0.65,1.052)*** | 0.851 (0.65,1.052)*** | 0.851 (0.733, 0.969)*** |
| Proportion of SC and ST households | 0.047 (-0.095,0.188) | 0.047 (-0.095,0.188) | 0.047 (-0.077, 0.170) |
| Proportion of Muslim households | 0.168 (0.027,0.309)* | 0.168 (0.027,0.309)* | 0.168 (0.011, 0.326)* |
| Proportion of poorest households | 0.227 (0.025,0.43)* | 0.227 (0.025,0.43)* | 0.227 (0.067, 0.387)** |
| Place of residence  (reference category: Urban) |  |  |  |
| Rural  2.hv025 | -0.133 (-0.2,-0.067)*** | -0.133 (-0.2,-0.067)*** | -0.133 (-0.205, -0.062)*** |
| Region (reference category: North) |  |  |  |
| Central  region 2 | -0.101 (-0.369,0.167) | -0.101 (-0.369,0.167) | -0.101 (-0.560, 0.357) |
| East  region 3 | 0.003 (-0.306,0.312) | 0.003 (-0.306,0.312) | 0.003 (-0.418, 0.423) |
| Northeast  region 4 | 1.1 (0.676,1.524)*** | 1.1 (0.676,1.524)*** | 1.010 (0.734, 1.465)*** |
| West region 5 | -0.039 (-0.431,0.353) | -0.039 (-0.431,0.353) | -0.0386 (-0.479, 0.402) |
| South | 0.185 (-0.231,0.601) | 0.185 (-0.231,0.601) | 0.185 (-0.180, 0.550) |
| Constant | 13.534 (13.016,14.052)*** | 13.534 (13.016,14.052)*** | 13.534 (13.020, 14.048)*** |
| Random part | Variance (95% confidence interval) | | |
| State | 0.109(0.052,0.226) | 0.109(0.052,0.226) | 0.109 (0.060, 0.198) |
| District | 0.076(0.053,0.11) | 0.076(0.053,0.11) | 0.077 (0.059, 0.100) |
| Primary sampling unit | 0.288(0.21,0.394) | 0.288(0.21,0.394) | 0.288 (0.228, 0.363) |
| Household | 2.214(1.576,3.109) | 2.214(1.576,3.109) | 2.214 (2.020, 2.427) |
| Residual | 5.365(4.846,5.939) | 5.365(4.846,5.939) | 5.365 (5.176, 5.560) |

**Reference:**

[1]        Carle AC. Fitting multilevel models in complex survey data with design weights: Recommendations. *BMC Medical Research Methodology*; 9. Epub ahead of print 2009. DOI: 10.1186/1471-2288-9-49.
