# Supplementary figures and images for: Community-level women’s education and undernutrition among Indian adolescents: A multilevel analysis of a national survey
Source: PLoS One. 2021 May 20;16(5):e0251427. doi: 10.1371/journal.pone.0251427 (PMC8136857; doi:10.1371/journal.pone.0251427)

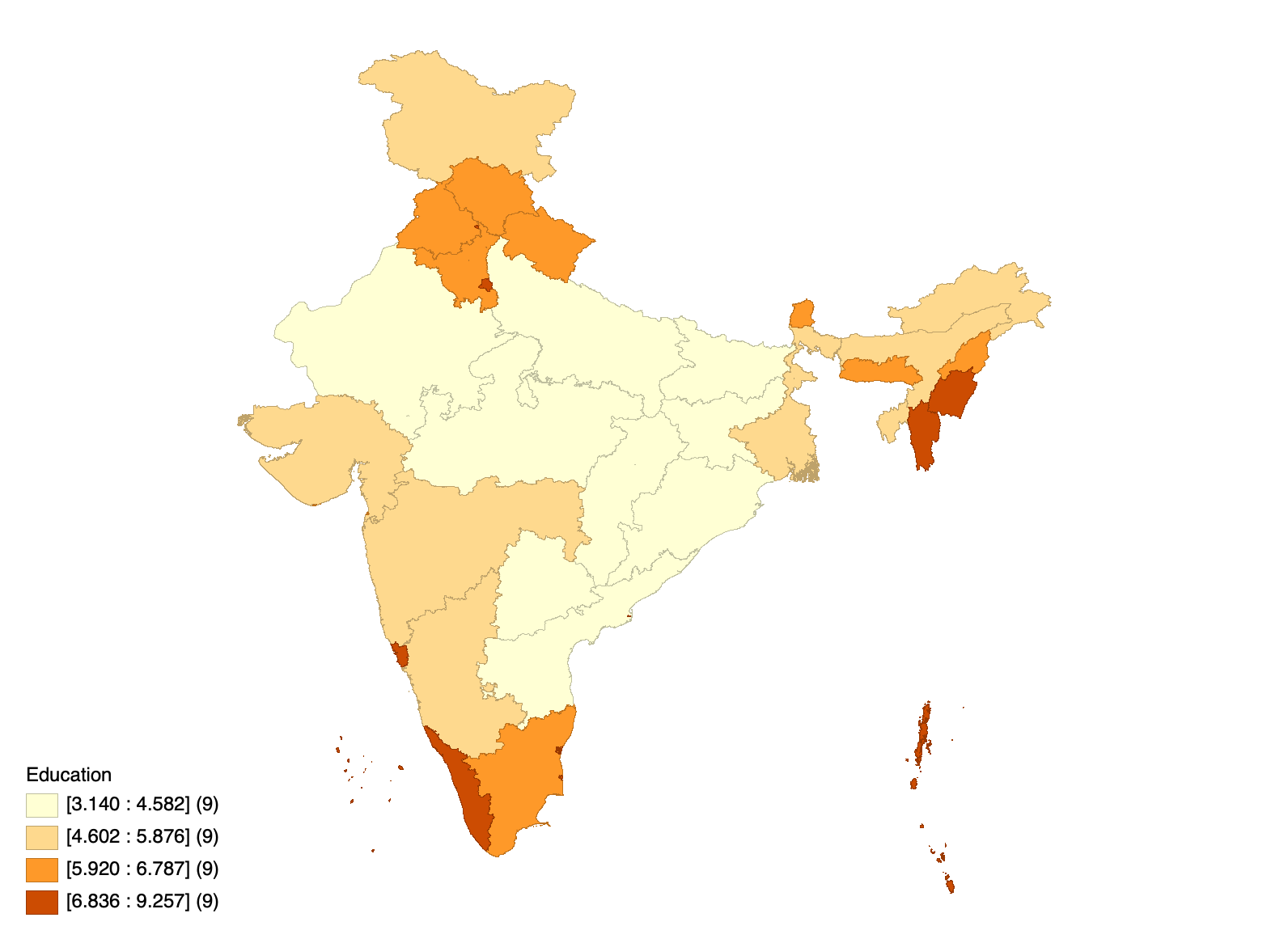

Supplement: S1 Fig — (TIF) [file pone.0251427.s004.tif]

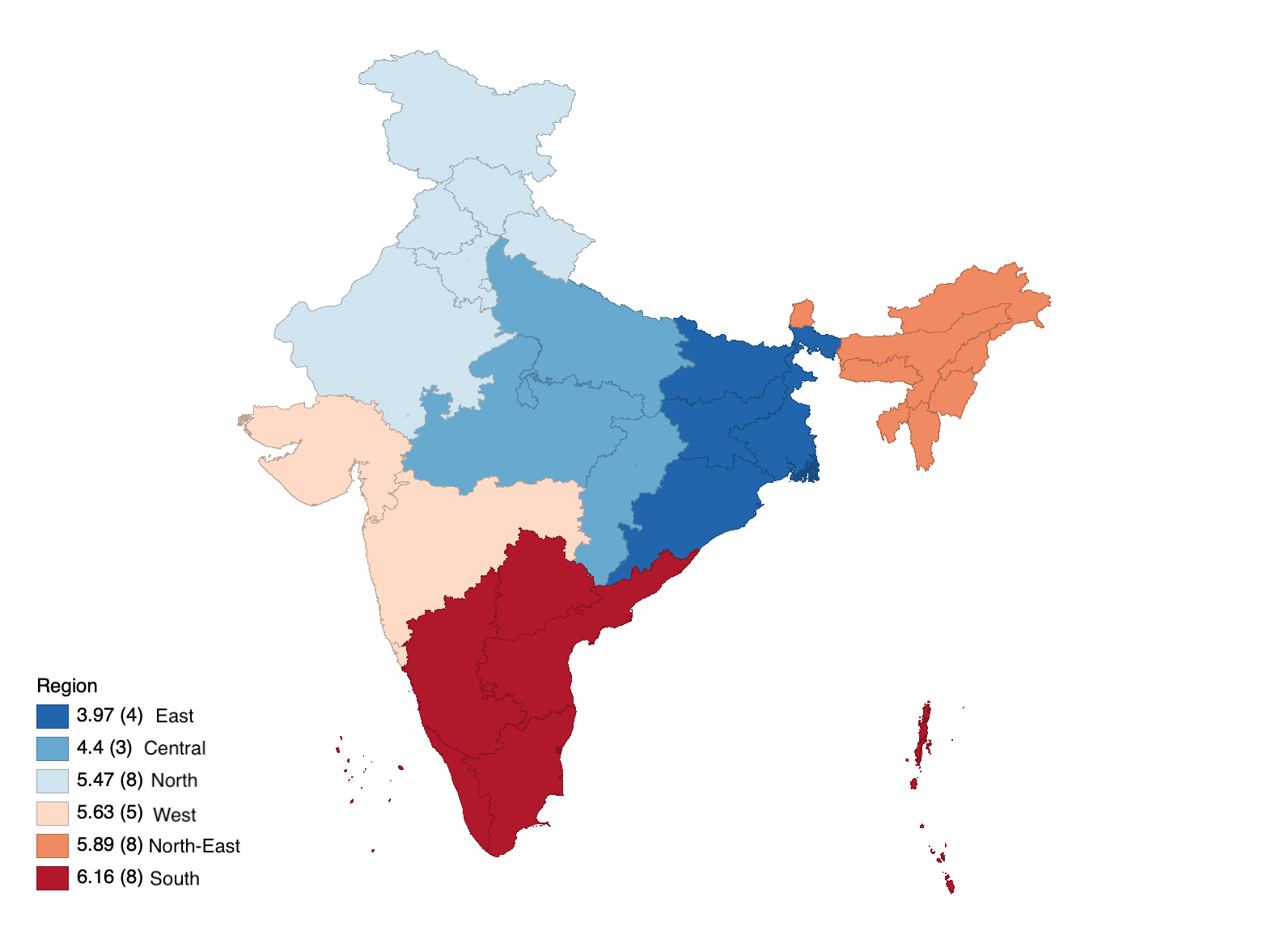

Supplement: S2 Fig — (TIF) [file pone.0251427.s005.tif]
